# Massive Lower Gastrointestinal Hemorrhage as a Complication of Severe Campylobacter Enteritis

**DOI:** 10.7759/cureus.24239

**Published:** 2022-04-18

**Authors:** Radhika Sharma, Barrett O Attarha, Kerolos Abadeer, Bruno Ribeiro

**Affiliations:** 1 Internal Medicine, University of Florida College of Medicine, Jacksonville, USA; 2 Gastroenterology, University of Florida College of Medicine, Jacksonville, USA; 3 Gastroenterology, University of Florida Health, Jacksonville, USA

**Keywords:** campylobacteriosis, severe enteritis, hematochezia, massive gastrointestinal bleed, diarrhea, lower gastrointestinal hemorrhage, campylobacter enteritis

## Abstract

Campylobacter enteritis is typically caused by *Campylobacter jejuni* or *Campylobacter coli* and is a major cause of diarrheal illness worldwide. Patients with Campylobacter gastroenteritis can be asymptomatic, but commonly present with a wide range of clinical symptoms including abdominal pain, diarrhea, vomiting, and occasionally self-resolving hematochezia. Although hematochezia can occur, acute massive lower gastrointestinal (GI) bleeding is a rare complication of Campylobacter gastroenteritis and should be considered as a possible differential diagnosis in the presentation of lower GI bleeds. We describe a unique case of a 48-year-old male who presented with massive lower GI bleeding requiring multiple transfusions and admission to the medical intensive unit; the patient was ultimately diagnosed with severe Campylobacter gastroenteritis.

## Introduction

Derived from the Greek words campylo meaning “curved” and bacter meaning “rod,” the Campylobacter species is composed of small, corkscrew Gram-negative rods that colonize mucous membranes of the reproductive and gastrointestinal (GI) tract of animals, particularly poultry [1,2]. Campylobacters are widely invasive and can be found to contaminate raw meat, both fresh and salt waters, and can survive for many weeks at a wide range of temperatures-this colonization allows for frequent animal-to-human transmission [2]. Generally, *Campylobacter jejuni* and *Campylobacter coli* are the two major causes of human disease and infection is acquired through either the consumption of undercooked meats or by the consumption of foods contaminated by contact with colonized meats [1,2].

The clinical manifestations of enteritis due to either *C. jejuni* or *C. coli* are similar and indistinguishable from enteritis caused by other bacteria such as Shigella or Salmonella [1]. It is important to note that some patients may present without any symptoms, however, asymptomatic infections are more common in resource-limited regions of the world where Campylobacter is hyperendemic, and high transmission rates in early childhood results in progressive immunity in older children and adults [3]. Typically, Campylobacteriosis presents with symptoms of cramping abdominal pain and acute-onset diarrhea [4]. One-third of patients may also experience prodromal symptoms of a febrile illness accompanied by rigors, generalized malaise, and delirium; these patients tend to have more severe disease [1,4]. Diarrheal symptoms may occur for about a week and can include up to 10 or more watery bowel movements a day and are usually self-limiting [4]. Hematochezia can be observed on the second or third day of diarrhea in about 15% of patients, however, massive GI hemorrhage is an extremely rare and life-threatening complication of Campylobacter enteritis [4,5].

## Case presentation

A 48-year-old male with a history of the cerebral vascular accident two years prior and hypertension presented to the emergency room complaining of five days of progressive worsening epigastric abdominal pain accompanied by severe diarrhea that began after eating Chinese food. The patient reported taking over-the-counter anti-diarrheal medications which initially improved his symptoms, however, he again became concerned when he began to have hematochezia about 24 hours prior to coming to the hospital. Upon further questioning, the patient denied any history of hemorrhoids, family history of colon cancer, or any issues with bowel function prior to presentation. He also denied the use of non-steroidal anti-inflammatory drugs and alcohol. His only medications were anti-hypertensives and a statin.

Investigation

**Figure 1:**
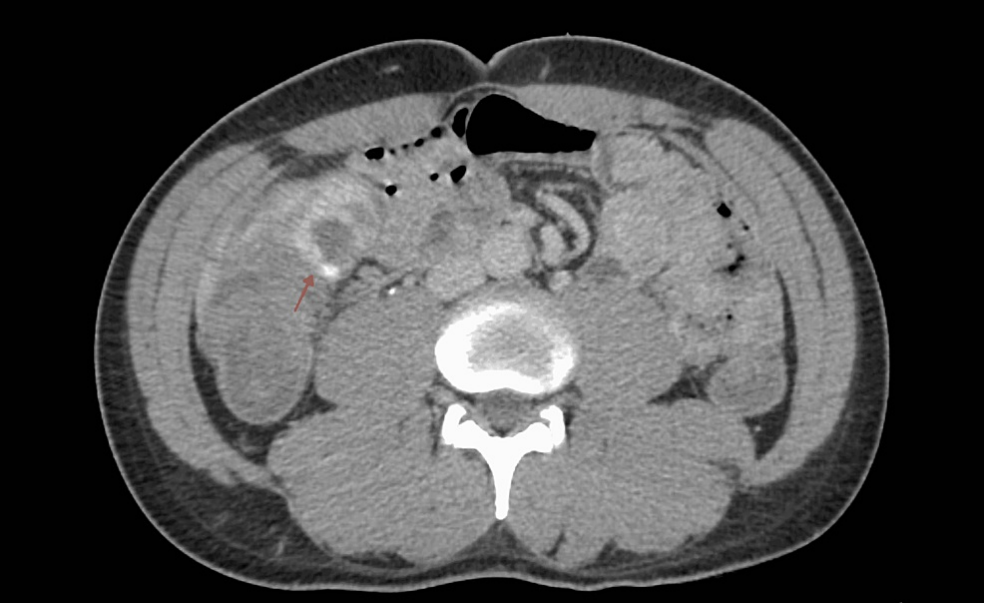
Computed tomography angiography acute gastrointestinal bleeding protocol revealing extravasation in the cecum and proximal ascending colon (red arrow).

Upon initial presentation, he was tachycardic to the 120s, hypotensive, and diaphoretic. Initial complete blood count and chemistry panels are shown below in Table 1. The patient had no history of anemia and his hemoglobin was normal two months prior. Lactic acid was found to be elevated to 4.1. While in the emergency department, the patient had multiple episodes of hematochezia. He was given one liter of lactated ringers, transfused two units of packed red blood cells (pRBCs), and was also started on intravenous pantoprazole. Vitamin K administration was deferred as the coagulation panel was within normal limits. The patient underwent Computed tomography angiography for acute GI bleeding which revealed extravasation in the cecum and proximal ascending colon (Figure 1). The patient was admitted to the medical intensive care unit (MICU) and the GI service was consulted along with interventional radiology for possible intervention. Upon arrival at the MICU, the patient continued to have persistent episodes of hematochezia associated with diaphoresis and hypotension.

**Table 1:** Complete blood panel two months prior to presentation vs. on emergency department presentation prior to any intervention

	Two months prior	On ED presentation
Hemoglobin (8-14 g/dL)	14	7.5
Hematocrit (40-54%)	41	22.5
Platelets (140-440 thou/cumm)	207	160
White Blood Cells (4.5-11 thou/cumm)	5.88	9.2

Treatment and outcome

**Figure 2:**
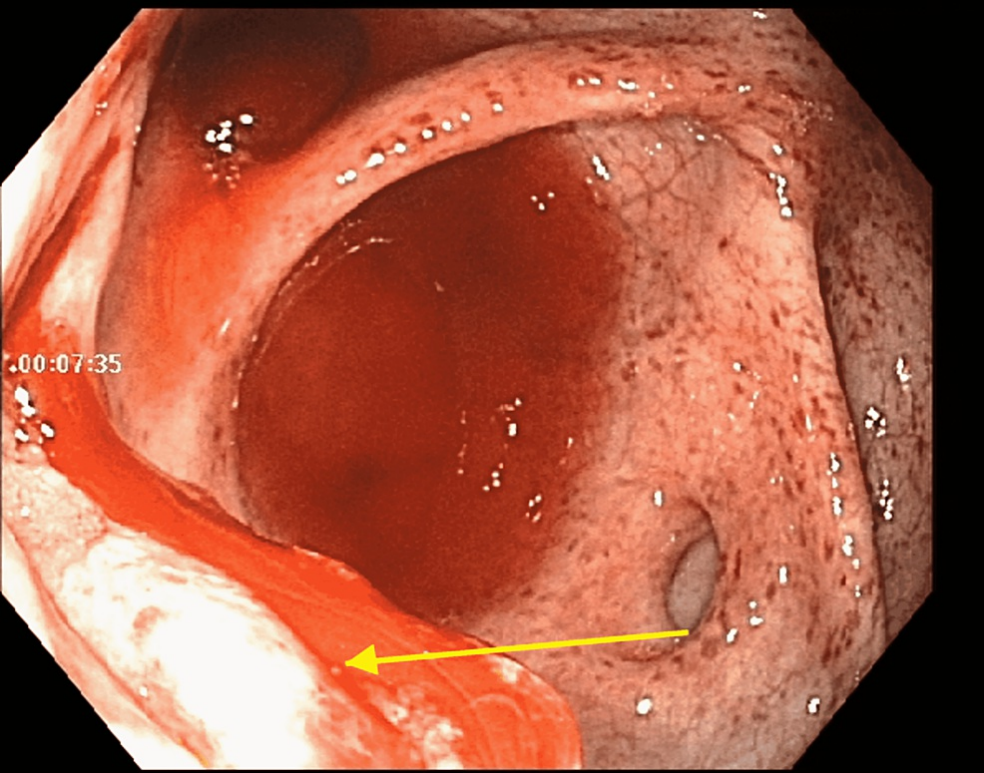
Colonoscopy revealing diffuse blood along the entire colon with mucosal abnormalities most prominent at the ileocecal valve.

Upon evaluation, the patient reported he had not had anything to eat in 12+ hours due to ongoing abdominal pain and the GI service decided to pursue bedside esophagogastroduodenoscopy (EGD) and colonoscopy. Colonoscopy revealed diffuse blood and blood clots along the entire colon (Figure 2). Additionally, mucosal abnormalities were visualized around the cecum and were most prominent at the ileocecal valve. No one source of active bleeding was identified and a clip was placed on the mucosal abnormalities near the ileocecal valve. Mucosal biopsies were also taken from the ileocecal valve area given the apparent abnormality. EGD was performed and was grossly normal with no signs of bleeding or evident pathology. The enteric pathogen nucleic acid amplification test (NAAT) which was ordered on admission resulted shortly after the completion of the EGD/colonoscopy and was positive for Campylobacter.

Oral azithromycin was started for treatment of Campylobacter infection for three days, which the patient was able to tolerate. The patient required two additional units of pRBCs and thereafter remained stable. He subsequently had formed bowel movements with no evidence of hematochezia or melena. Other than the medications mentioned above, no other medications were used in the therapeutic management of the patient. Biopsy results from the ileocecal valve showed colonic mucosa with acute colitis without evidence of chronicity or granulomas (nonspecific inflammation).

The patient had a total four-day hospital course. On stabilization and resolution of symptoms, the patient was discharged home. The patient’s hemoglobin was stable at 7.9 g/dL at the time of discharge. The patient was scheduled for outpatient follow-up with the gastroenterology clinic, however, was lost to follow-up.

## Discussion

Massive GI bleeding has been reported as a manifestation of parasitic intestinal infections however, it is an extremely rare complication in Campylobacteriosis [5,6]. Self-limited episodes of hematochezia are not uncommon in Campylobacter enteritis and are caused by invasive intestinal disease leading to mucosal damage and diffuse inflammation [7]. In our patient, the colonoscopy revealed extensive blood and blood clots throughout the entire colon with no one specific source of active bleeding.

Massive lower GI bleeds are categorized by bright red blood per rectum, usually occur in patients 65 years and above with multiple medical comorbidities, and have a mortality as high as 21% [8]. Patients may present with hemodynamic instability with systolic blood pressures equal to or less than 90 mmHg, tachycardia with heart rate greater than or equal to 100 beats/min with symptoms of altered mental status, diaphoresis, lightheadedness, dizziness, or syncope [8]. The majority of massive lower GI bleeds are attributable to angiodysplasias and diverticulosis, however, a thorough differential diagnosis should include inflammatory, neoplastic, congenital, and infectious etiologies including specifically Campylobacter enteritis [8,9].

Management of massive lower GI bleeding involves immediate evaluation and stabilization of the airway, establishment of access with two large-bore peripheral intravenous drips, and continuous cardiopulmonary monitoring [8]. Early resuscitation efforts should be initiated with intravenous infusions of crystalloids and consideration of transfusion of blood products guided by history and physical, lab work (complete blood count, metabolite panel, liver function tests, lactic acid, and coagulation panel), and further clinical course [8]. Ultimately, GI evaluation with EGD and/or colonoscopy will guide treatment. Although the time of colonoscopy continues to remain controversial, most guidelines suggest colonoscope evaluation to be performed within 24 hours of admission following adequate bowel preparation [8]. As for treatment for symptomatic Campylobacter enteritis and prevention of further complications, antibiotic therapy with erythromycin and ciprofloxacin are effective in 90% of cases and should be initiated in patients with severe disease manifestations [5].

## Conclusions

Campylobacter enteritis is a major cause of diarrheal illness worldwide. Patients with Campylobacter gastroenteritis can be asymptomatic, but commonly present with a wide range of symptoms including abdominal pain, diarrhea, vomiting, and occasionally self-resolving hematochezia. Massive hematochezia is a rare complication of Campylobacter gastroenteritis and should be considered as a possible differential diagnosis in the presentation of massive lower GI bleeds. Management and treatment involve immediate evaluation and stabilization along with EGD and/or colonoscopy and further symptomatic control with antibiotic therapy.
